# Post-Dural Puncture Headache: Haunts the Anaesthetist

**DOI:** 10.5812/kowsar.22287523.2849

**Published:** 2012-01-01

**Authors:** Seza Apiliogullari, Jale Bengi Celik

**Affiliations:** 1 Department of Anesthesia and Intensive Care, Selcuk University, Selcuklu Medical Faculty, Konya, Turkey

**Keywords:** Post-Dural Puncture Headache, Anesthesia, Spinal

Dear Editor,

We read with great interest study conducted by Mosaffa et al. (1), designed to compare the rate of post-dural puncture headache (PDPH) in median and paramedian approaches in spinal anesthesia in the September issue of Anesthesiology and Pain Medicine.

Spinal anaesthesia has been used in patients from preterm neonates to aged over a hundred years. At present it is a gold standard for many different procedures. We are using spinal anaesthesia not only as an alternative to general anaesthesia but also as the first choice for suitable surgery in both children and adults with or without co-morbidity. We believe that main restrictive complication is named post-dural puncture headache which haunts the anaesthetist and the surgeons who face the complaint next morning after spinal anesthesia. Studies reported that small gauge pencil point needles leads to significantly less incidence of PDPH in children (2) and adults (3). Controversy still exists about the use of thin and pencil point spinal needles since they seem to be difficult to handle, resulting in a reduced success rate and increased cost. We believe that future trials must be planed to be alternative to small gauge pencil point needles like Mosaffa et al. (1) study.

We would like to ask to author “ why haven’t they chosen parturient as subjects for evaluated PDPH on the contrary many of reported studies which were evaluted PDPH have taken parturient as subjects who are at a particularly high risk of PDPH?”
